# Emotion Understanding in Bilingual Preschoolers

**DOI:** 10.3390/bs12040115

**Published:** 2022-04-18

**Authors:** Daria Bukhalenkova, Aleksander Veraksa, Margarita Gavrilova, Natalia Kartushina

**Affiliations:** 1 Department of Psychology, Lomonosov Moscow State University, Moscow 119991, Russia; d.bukhalenkova@inbox.ru (D.B.); veraksa@yandex.ru (A.V.); 2 Psychological Institute of Russian Academy of Education, Moscow 125009, Russia; 3 MuliLing, Institute for Linguistics and Scandinavian Studies, University of Oslo, 0315 Oslo, Norway; natalia.kartushina@iln.uio.no

**Keywords:** emotion understanding, bilingualism, non-verbal intelligence, culture, language

## Abstract

The effects of bilingualism on child development have been extensively examined in last decades. Research reveals that simultaneous use of two or more languages affects child’s language development, cognitive and social skills. The current study focuses on the so-far understudied theory of emotion understanding in bilingual children. A cohort of 593 bilingual and monolingual 5–6-year-olds took the Russian version of the Test of Emotion Comprehension (TEC) that assesses three components of emotion understanding: emotion understanding of external causes of emotions, reflective causes of emotions; and mental causes of emotions. Our results revealed no group differences between overall emotion understanding and understanding of external and reflective causes of emotions. However, monolingual children had a slightly better understanding of mental causes of emotions compared to bilingual children, when controlling for age, gender, and non-verbal intelligence. These results suggest that children growing up in bilingual environments might require more time and/or language/culture exposure to master the ability to understand mental causes of emotions, taking into account cultural differences, as well as the semantic and lexical differences in emotion labelling and emotion expression in each language.

## 1. Introduction

The effects of bilingualism on child development have been extensively examined in last decades, likely due to the globalization and increasing mobility in today’s world. Nowadays, it is common, in a variety of countries, that children use two or more languages from early childhood. A dual-language learner is a person who acquires two or more languages simultaneously, and learns a second language while continuing to develop his or her first language [1]. Yet, as Bhatia mentions, bilingualism has broader effects beyond language, including the learner’s individual, social, psychological and academic life [2]. Research indicates that simultaneous use of two or more languages can affect a child’s development of cognitive [3,4,5,6] and social skills [7], in addition to well-being [8]. However, very few studies have addressed the development of emotions in bilingual children [9,10,11,12]. The current study fills this gap in research on emotion understanding in bilingual preschool children.

### 1.1. Multilingualism in Russia

In Russia, research on bilingualism has important practical significance as, historically, many nationalities have been intertwined geographically, economically and culturally, leading to the current multinational state. Notably, 190 ethnicities live on the territory of the country, among which approximately 80% are Russian. Out of the 85 states/regions of the Russian Federation, 22 are national Republics that account for 28.6% of the de facto territory of Russia and are home to over 18% of the country’s population [13]. Russian is the state language, but many of the national Republics have their own “state” language that is used in conjunction with Russian. In addition to Russian, the use of local languages in minority areas is established by law (e.g., in the Republics of Altai, Bashkortostan, Tatarstan, Chuvashia, the Republic of Sakha (Yakutia), and the Republics of the North Caucasus). According to the ratio of language use in multilingual Russian republics, two types of bilingualism can be distinguished: Russian dominant, when listeners speak only Russian or have some knowledge of the autochthonous/ethnic language, which is typical for the urban population; and ethnic dominant, when listeners speak the language of the native ethnic group, but knowledge of Russian may be poor and/or incomplete (typical for rural populations) [14]. A more balanced command of Russian and regional languages is found in the Republics where the regional language has a state status (e.g., Tatarstan, Sakha (Yakutia), etc.). In such Republics, kindergartens and schools provide care and teach children in Russian and in the language of the native ethnic group [15]. To conclude, on the territory of the current Russian Federation, at least 20% of children grow up in a relatively balanced bilingual environment [13].

### 1.2. Emotion Understanding at Preschool Age

Emotion understanding is defined as the ability to understand the nature, causes, and consequences of one’s own emotions, and the emotions of others. Understanding emotions includes recognition, description, explanation, prediction and control of emotion expression in everyday life [16]. Understanding emotions is part of a child’s social and emotional development, and one of the components of emotional competence [17,18]. Emotional competence includes three abilities that help children build relationships with others and cope with difficulties in communication: (a) the ability to recognize and understand one’s own and others’ emotional expressions and experiences [19,20]; (b) emotional expressivity, or rather the ability to experience and express a range of emotions, facilitating social interaction; and (c) emotion regulation, which usually refers to the ability to manage, control and correct emotional reactions to adjust to and cope with situations [21,22].

The cultural–historical approach to child development places children’s emotional and social competence at the center of development. Vygotsky argued that emotions are internal determinants of thought and have significant influence on children’s cognitive development [23]. Later on, Vygotsky’s pupils associated emotion understanding with the development of “the emotion anticipation and the anticipation of the consequences of a child’s actions” [24]. Zaporozhets paid special attention to emotion development in preschool- and school-aged children, and proposed that emotional anticipation arises as a result of a child’s internal orientation and research activity. Thus, emotion anticipation is directly connected to the child’s interaction with the environment. Orientation and research activity lead to the establishment of a functional system that combines affective and cognitive processes [24].

Current approaches to the study of emotion understanding, stemming from the results of empirical research, offer a stage model for children’s emotion development [25,26,27,28,29]. Emotion understanding develops progressively during preschool age and becomes the foundation for the child’s further social–emotional and cognitive development. According to the theoretical model by Harris and Pons [28], the main stages in the development of emotion understanding occur in the preschool years. The first stage corresponds to 3–5 years of age, when children learn to understand the external causes of emotions (recognition of the basic emotions of others by facial expressions and understanding the influence of external circumstances and desires on emotions). The second stage occurs between 5 and 7 years of age, when children gradually start understanding that personal beliefs and memories can evoke different emotions, and also that some emotions can be hidden. Then, at the third stage, 7–9-year-old children learn to regulate their emotions using cognitive strategies, learn that moral rules can influence emotions, and that emotional states can be contradictory [30,31,32].

Individual differences in children’s emotion understanding have been attributed to differences in family environment, such as parents’ education [27,33], parents’ attitudes towards emotions [34], parents’ ability to accurately assess children’s emotion understanding skills [33], and parents’ emotional vocabulary [35,36]. Other studies have attributed differences in children’s emotion understanding to differences in cognitive and affective skills [26,37]. To conclude, the studies conducted so far represent a significant step forward towards our understanding of the development of emotion understanding in children; yet, research on emotion development in bilingual children is sparse and, to our knowledge, only one study by Weimer and Gasquoine [11], so far, has addressed emotion understanding in bilingual children.

### 1.3. Emotion Understanding and Bilingualism

The ability to understand emotions, to a large extent, develops through an interaction with others within a given cultural context, where language serves, many times, as the main vehicle for the interaction [38,39,40]. Language enables a child to acquire important cultural norms shared within a given society [41]. For example, social interaction and language use play a key role in children’s acquisition of emotion understanding [42]. Bilingual children acquire two languages and, often, language learning is carried within two cultural contexts. LaFromboise and colleagues proposed that successful assimilation to a bicultural environment involves, among others, knowledge of cultural beliefs and values in each culture, positive attitudes towards both cultures, bicultural efficacy, communication ability, role repertoire, and a sense of being grounded in both cultures [42]. Given that expectations and values for emotional expression vary between cultures, a bilingual child needs to learn at least two sets of cultural norms, values, and expectations regarding how emotions should be handled. The child does not only need to learn and distinguish between these sets of cultural expectations, but also to understand the specific context of each individual situation to respond appropriately and adapt to the emotions of others. Cheung and colleagues suggest that bilingual children pay more attention to emotions than their monolingual peers [43]. Therefore, it is of particular practical and theoretical importance to examine emotion understanding in bilinguals.

Research on emotion development in bilingual children is sparse and the results are not conclusive. For instance, Weimer and Gasquoine [11] examined emotion understanding in Mexican American young children and reported no differences between unbalanced (one language is dominant) and balanced bilinguals. Janus and Bialystok [12] revealed no significant differences in emotional processing between bilingual and monolingual 8–11-year-old typically developing children [12]. Research on emotion and autobiographical memory in bilinguals, on the other hand, has shown that emotional events were reported differently in oral narratives in the mother tongue as compared to the second language [44,45]. Bilinguals were better at conveying the emotional experience in their mother tongue than in their second language, even though the content of the event was narrated equally well/fluently in both languages [45]. The researchers noticed that parents of bilingual children were more likely to use their mother tongue to express emotions and feelings towards their child, which might explain the results [45].

Pavlenko [45] has thoroughly examined the connection between language and emotion in bilinguals [45]. She supports a constructionist view of emotion and argues that emotions themselves and the way they are expressed are inseparable from the language. Bilinguals, therefore, could face difficulties in experiencing and expressing emotions across languages because of the differences in how emotions are coded between languages (e.g., as processes or as states), among other causes (e.g., their proficiency, experience, etc.). Bilinguals must acquire a fair amount of knowledge, in both languages, to be able to adequately express and decode emotions in a bicultural environment (e.g., emotion vocabulary, choice of pronouns, pragmatic knowledge), as noted by Pavlenko [45]. However, research comparing bilinguals’ emotion understanding to that of monolinguals is still sparse. The current study aims to fill this knowledge gap on emotion development in bilingual children and examines emotion understanding in bilingual Russian preschoolers.

### 1.4. The Current Study

The aim of the present study was to compare emotion understanding in 5–6-year-old Russian monolingual and bilingual children, controlling for age, gender and non-verbal intelligence, which are known to affect emotion understanding [27,37,46]. We expected that a divergence in attitudes towards emotions and how they are expressed across cultures would have a negative impact on the development of emotion understanding skills in bilingual children. We addressed our aim by examining the experimental data from three geographically and culturally distinct Russian regions.

## 2. Materials and Methods

### Participants

The recruitment process was based on an existing agreement between Lomonosov Moscow State University (MSU) and a number of kindergartens in three geographically and linguistically distant areas of the Russian Federation: the Moscow region, and the republics of Tatarstan and Sakha (Yakutia). Parents received emails from the educational institutions that their child attended to informing them of the opportunity to participate in the study. Originally, a total of 900 parents were invited to participate in the study, but only 593 parents gave their informed consent and agreed to allow their child to participate in the study. After obtaining the informed consent from the parents, we sent them a link to fill in a short electronic demographic form indicating the age and gender of their child, their education, and the characteristics of the linguistic environment in which their child had been growing up. All children were individually assessed in their kindergarten. On average, the test lasted between 15 and 20 min. The study and the consent forms were approved by the Ethics Committee of Faculty of Psychology at Lomonosov Moscow State University (the approval No: 2020/61).

The final sample consisted of 593 typically developing children (46.9% boys) aged 5–6 years, with a mean age of 5.21 years (SD = 0.38). The children were attending public kindergartens in three regions of Russia. The Republic of Tatarstan and the Republic of Sakha (Yakutia) represented the regions where children grew up in bilingual environments and the city of Moscow represented a predominant monolingual region (see Table 1 for details). Most mothers had a university degree (83.5%) or a diploma in a secondary professional education (11.7%); 2.6% of mothers had a compulsory school diploma or an incomplete higher education, and 2.2% of mothers had an academic degree. The socioeconomic statuses of the families (SES) varied, with middle-income families comprising 75.1%, low-income families comprising 12.6%, and high-income families comprising 12.2%.

The children were assigned to the monolingual (*n* = 319) or bilingual (*n* = 278) group based on the parental responses to the language questionnaire. The questionnaire identified which languages the child knew, which language(s) the child spoke more often, which languages were spoken to the child at home, and which languages were spoken to the child in her kindergarten. In the monolingual group, children always (or more than 80% of the time) spoke one language, and the same language was spoken at home and in kindergarten. In the bilingual group, children used two languages (e.g., typically, one language at home and another one in kindergarten), with a minimum of 20% of exposure to the second language.

## 3. Materials

### 3.1. Emotion Understanding

The Russian version of the Test of Emotion Comprehension (TEC) [47,48] was used to assess children’s (emotion understanding) EU. The Russian version of the TEC was successfully adapted and validated for use in a Russian sample [46]. The test material was an illustrated book with simple stories. For each story, there were four drawings with different facial expressions. After that, the experimenter read the story to the child, and the child was asked to choose ‘the picture that represented the feelings of the character in the story’. The answers were non-verbal. The TEC assesses three hierarchical levels of emotion understanding: external, mental, and reflexive. The external level focuses on the ability to recognize emotions, to understand the external causes of emotions, and the impact of desires on emotions. The mental level concerns understanding the role of beliefs and memories on emotions, as well as understanding of hidden emotions. The reflexive level is the most complex and evaluates understanding of mixed feelings, the possibilities of emotion regulation via cognitive strategies, and the influence of moral self-reflective rules on emotions. For each test the score could range between 0 and 3. The child’s overall score in emotion understanding was expressed by the sum of scores in each level; therefore, the final score could vary between 0 and 9.

### 3.2. Non-Verbal Fluid Intelligence

Non-verbal fluid intelligence was evaluated using the Russian adaptation of Raven’s Colored Progressive Matrices (CMPM) [49]. The task involved completing matrices of patterns and figures by deducing which of the four options completed the matrix correctly. We counted the number of correct answers until the child made four mistakes in a row, then the test was stopped. The final score could vary between 0 and 36.

## 4. Results

Jamovi software, version 1.0.7.0 (The jamovi project) was used for all the analyses run in the current study. First, an independent sample *t*-test was performed to confirm that the monolingual and bilingual groups did not differ significantly in age, non-verbal intelligence and gender. The independent samples *t*-test showed that monolingual and bilingual groups did not differ significantly in age (595) = 1.8591, *p* = 0.063, non-verbal intelligence (595) = 1.0582, *p* = 0.290, or gender (591) = −0.0539, *p* = 0.957 (see Table 2).

The next step in the analyses was to examine differences between bilingual and monolingual groups (independent variable) for their general level of emotion understanding (TEC emotion) and subsequent hierarchical levels (TEC external, TEC mental, and TEC reflective). Four separate general linear models (GLM) were built to assess group differences on each level of emotion understanding, controlling for age, gender, non-verbal intelligence, and the interaction effect between the groups and non-verbal intelligence.

### 4.1. General Ability to Understand Emotions

GLM was performed to compare the general ability of monolingual and bilingual children to understand emotions (TEC emotion) with the following covariates: age (continuous in years), non-verbal intelligence (continuous), gender (categorical with two levels), and the interaction between the groups and non-verbal intelligence. An ANOVA Omnibus test indicated that the model described the data well: F (5, 590) = 19.06, *p* < 0.001, η^2^*p* = 0.115. Regarding the general ability to understand emotions, there was a highly significant effect of age (F (1, 590) = 16.65, *p* < 0.001, η^2^*p* = 0.028) and non-verbal intelligence (F (1, 590) = 42.19, *p* < 0.001, η^2^*p* = 0.067). Older children were better in general emotion understanding (M = 5.16, SD = 0.089) than younger children (M = 4.64, SD = 0.089). Children with high non-verbal intelligence performed better (M = 5.31, SD = 0.089) than children with lower non-verbal intelligence (M = 4.48, SD = 0.089). There were no significant main effects or interactions between gender, group, or groups and non-verbal intelligence (*p* > 0.1).

### 4.2. External, Mental and Reflective Levels of Emotion Understanding

Similar GLM was performed to compare the ability of monolingual and bilingual children to understand external causes of emotions (TEC external). An ANOVA Omnibus test indicated that the model described the data well (F (4, 590) = 14.25, *p* < 0.001, η^2^*p* = 0.089). A significant effect of age (F (1, 590) = 20.74, *p* < 0.001, η^2^*p* = 0.034), non-verbal intelligence (F (1, 590) = 20.41, *p* < 0.001, η^2^*p* = 0.034) and gender (F (1, 590) = 6.36, *p* = 0.012, η^2^*p* = 0.011) was revealed. Children with high non-verbal intelligence showed a better understanding of external causes of emotions (M = 2.60, SD = 0.039) than children with lower non-verbal intelligence (M = 2.44, SD = 0.039). The older children showed a better understanding of external causes of emotions (M = 2.60, SD = 0.04) than the younger children (M = 2.34, SD = 0.04). Girls displayed a better understanding of external causes of emotions (M = 2.55, SD = 0.04) than boys (M = 2.41, SD = 0.04). There were no significant main effects or interactions between group (bilingual and monolingual) and non-verbal intelligence.

**Figure 1:**
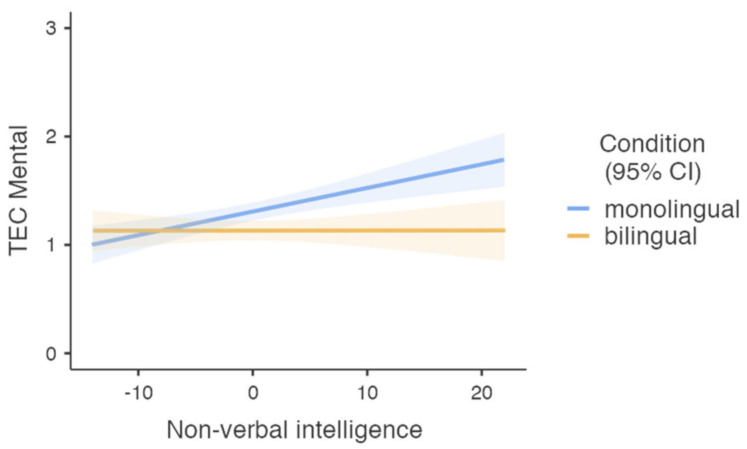
Ability to understand mental causes of emotions in monolingual (*n* = 319) and bilingual (*n* = 277) children with different levels of non-verbal fluid intelligence. Colored shades represent the confidence intervals. Note: non-verbal intelligence scores presented as Z scores.

A similar separate GLM was performed to compare monolingual and bilingual children’s ability to understand mental causes of emotions (TEC mental). An ANOVA Omnibus test indicated that the model described the data well (F (5, 590) = 8.71, *p* < 0.001, η^2^*p* = 0.069). There was a significant main effect of group (F (1, 590) = 7.7, *p* < 0.006, η^2^*p* = 0.013), age (F (1, 590) = 13.08, *p* < 0.001, η^2^*p* = 0.022), and non-verbal intelligence (F (1, 590) = 7.10, *p* < 0.008, η^2^*p* = 0.012). Monolingual children showed a better ability to understand mental causes of emotions (M = 1.31, SD = 0.043), as compared to their bilingual peers (M = 1.13, SD = 0.046). Older children performed better (M = 1.33, SD = 0.045) than younger children (M = 1.10, SD = 0.045), and children with higher non-verbal intelligence performed better (M = 1.30, SD = 0.044) than children with lower non-verbal intelligence (M = 1.13, SD = 0.045). However, the effect size was relatively small: Cohen’s d = 0.20. There was also a significant interaction between group and non-verbal intelligence (F (1, 590) = 7.10, *p* < 0.008, η^2^*p* = 0.012). As revealed in Figure 1, the ability of monolingual children to understand the mental causes of emotions was modulated by non-verbal intelligence. That is, higher non-verbal intelligence was associated with better understanding of mental emotions and vice versa. However, in bilingual children, this ability was stable and did not change with non-verbal intelligence scores.

**Figure 2:**
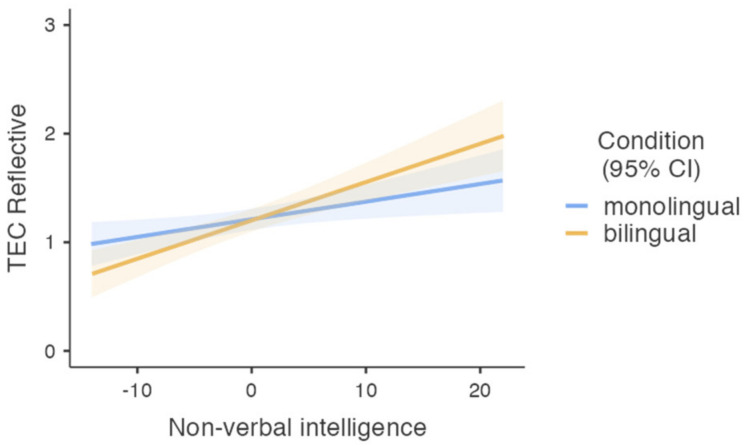
Ability to understand reflective causes of emotions for monolingual (*n* = 319) and bilingual (*n* = 277) children with different levels of non-verbal fluid intelligence. Colored shades represent the confidence intervals. Note: non-verbal intelligence scores presented as Z scores.

Finally, a similar GLM was performed separately to compare monolingual and bilingual children’s ability to understand reflective causes of emotions (TEC reflective). An ANOVA Omnibus test indicated that the model described the data well (F (45, 590) = 6.61, *p* < 0.001, η^2^*p* = 0.089). There was a significant effect of non-verbal intelligence on children’s ability to understand reflective causes of emotions (F (1, 590) = 20.41, *p* < 0.001, η^2^*p* = 0.034). Children with high non-verbal intelligence showed better understanding of reflective causes of emotions (M = 1.41, SD = 0.05) than children with lower scores of non-verbal intelligence (M = 1.01, SD = 0.06). There were no significant main effects of group (bilingual and monolingual), age, or gender (*p* > 0.1). However, the interaction between group and non-verbal intelligence was significant (F (1, 590) = 4.07, *p* = 0.044, η^2^*p* = 0.007). As illustrated in Figure 2, for both groups, higher non-verbal intelligence was associated with better understanding of the reflective causes of emotions, but the effect of non-verbal intelligence was stronger in bilingual children.

Note that all significant effects remained when we adjusted the *p*-value for multiple comparisons using the Bonferroni method (*p* = 0.0125).

## 5. Discussion

The aim of the current study was to investigate whether simultaneous acquisition of two languages impacted preschoolers’ emotion understanding when controlling for age, gender, and non-verbal intelligence. Overall, the results revealed no group differences, however, bilingual children demonstrated significantly lower results in understanding the mental causes of emotions compared to monolingual peers, when controlled for age, gender, and non-verbal intelligence. Our results showed, in addition, that while monolinguals’ capacity to understand mental causes of emotions increased with higher non-verbal intelligence, bilinguals’ performance was steadily, i.e., independent from non-verbal intelligence. To conclude, the results provide only partial support for the hypothesis that simultaneous acquisition and use of two or more languages are associated with a weaker ability to understand emotions [45]. Bilingual children displayed poorer performance only in the understanding of mental causes of emotions, and they performed similarly well (to monolingual children) in all other emotion understanding tasks. No differences were found between monolingual and bilingual children in the general ability to understand emotions, or in the understanding of external causes of emotions or reflective causes of emotions.

We consider there to be three possible interpretations of these results. The first possible explanation concerns the bicultural specificity of the social context in which a bilingual child acquires information about the nature and causes of emotions [49]. According to LaFromboise et al. [50], a bicultural person needs to master both sets of cultural expectations and navigate them well, successfully reading culture-specific contextual cues. Participants in this study were residents of three distinct regions of Russia with considerable cultural, religious and historical differences, which might have affected approaches to emotion socialization. Potential inconsistencies in cultural attitudes regarding emotions and their expression can weaken or delay bilingual children’s understanding of the mental causes of emotions in each specific culture, even in children with high non-verbal intelligence. Developing the ability to accurately decode emotions in a bicultural environment may require additional time for bilinguals, compared to monolingual preschoolers [27].

The second reason concerns cross-language differences in the linguistic properties of emotion-related words. There are numerous instances where the name of an emotion in one language does not have a direct translation in another language. Alekseeva [51] analyzed the emotion words that are used in Russian and Yakut languages (two of the main languages in the current study). She came to the conclusion that the lack of direct analogues between Russian and Yakut emotion words was possibly due to the common tradition in Yakut culture to use symbolic expressions to describe emotional states [51]. Thus, various concepts, including the names of emotions and feelings, have been historically formed in the Yakut culture and language on the basis of ritual symbolism [52].

The third explanation attributes bilingual children’s poorer performance in emotion understanding to a general reduction in language exposure and poorer language skills, as compared to monolingual peers. Previous studies have shown that bilingual children experience low verbal fluency, reduced lexical access, and lower passive vocabulary in both languages [53,54,55,56]. In other words, some of the cognitive and language processes that “serve” emotion understanding may be lagging in bilingual children compared to their monolingual peers [43], which may affect their emotion understanding. On the other hand, emotion understanding, to a large extent, takes place in a communicative setting and in interactions [57,58]. Most communication situations involve the use of a language [38,39,40]. Therefore, a child’s lack of knowledge and/or exposure to the language that is used for communication, or a difficulty to switch quickly between languages, may reduce the child’s engagement in an interaction with peers and adults. Nonetheless, considering the small effect size on the understanding of mental causes of emotions, and the absence of group difference in the general ability to understand external and reflective emotions, we suggest that, in general, growing up in bicultural and bilingual environments in a Russian context does not hinder emotion understanding in Russian preschool bilingual children.

### Limitations

These study results must be interpreted in the context of some limitations. First, the sample of the study was limited to three Russian regions. Future studies need to replicate the results of the current study with more diverse samples in order to obtain more representative samples and draw stronger conclusions about differences. Second, the study did not collect data on children’s language proficiency and age of acquisition, and the test was run in only one language, i.e., Russian; future research must consider the role of the type of bilingualism and language proficiency in emotion understanding. Despite the limitations mentioned above, the present large-scale study contributes to the field of emotion development in bilingual children by showing evidence for only a slight negative impact of bilingualism on children’s understanding of mental causes of emotions. The other two components of the evaluation, as well as the general emotion understanding score, did not differ from monolingual peers, even when controlling for age, sex, and non-verbal intelligence.

## 6. Conclusions

This paper presents important contribution to the field of emotion understanding in bilingual children. Specifically, bilingual children showed lower results in the understanding of the mental causes of emotions compared to monolingual peers; yet, no group differences were revealed in overall emotion understanding. These findings reiterate the importance of considering language and culture as important factors in terms of child experiences and development. The current results can be useful in the process of decision-making regarding educational policies in countries with multicultural populations, can be considered in psychological adaptation to prepare bilingual children for school, and can be useful for the development of educational programs and for the formation of relevant competencies in teachers and psychologists. More research and from a variety of linguistic and cultural backgrounds is needed, as adjusting educational policies to bilingual realities is becoming an acute priority in countries with multicultural and migratory populations.

## Figures and Tables

**Table 1:** Bilingual and monolingual children in the final sample.

Region	Group		*n*	Age	Girls (%)
	Monolingual	Bilingual			
Republic of Tatarstan	92	94	186	5.28 (±0.36)	59.1
Moscow	182	24	206	5.20 (±0.45)	49.5
Republic of Sakha (Yakutia)	43	160	203	5.16 (±0.30)	50.7

**Table 2:** Description statistics for study variables.

	Group	*n*	Mean	Median	SD
Age	monolingual	319	5.24	5.25	0.423
	bilingual	278	5.18	5.17	0.315
Non-verbal intelligence	monolingual	318	14.30	13.00	7.929
	bilingual	277	13.62	13.00	7.554
TEC External	monolingual	319	2.49	3.00	0.677
	bilingual	278	2.46	3.00	0.733
TEC Mental	monolingual	319	1.33	1.00	0.821
	bilingual	278	1.12	1.00	0.726
TEC Reflective	monolingual	319	1.21	1.00	0.842
	bilingual	278	1.19	1.00	0.962
TEC Emotion	monolingual	319	5.03	5.00	1.575
	bilingual	278	4.77	5.00	1.639

Note: During the assessment, two children refused to complete the non-verbal intelligence test. For this reason, there were 277 children for this task.

## Data Availability

The data presented in this study are available on request from the corresponding author. The data are not publicly available due to the details of ethical permission for research.
